# Prognostic Value of Albumin-to-Alkaline Phosphatase Ratio in Hepatocellular Carcinoma Patients Treated with Liver Transplantation

**DOI:** 10.7150/jca.39615

**Published:** 2020-02-03

**Authors:** Hui Li, Li Wang, Liang Chen, Hui Zhao, Jianye Cai, Jia Yao, Jun Zheng, Yang Yang, Genshu Wang

**Affiliations:** 1 Department of Hepatic Surgery and Liver Transplantation Center, the Third Affiliated Hospital of Sun Yat-sen University, Guangzhou, Guangdong, 510630, China.; 2 Department of Liver Surgery, Liver Transplantation Division, West China Hospital, Sichuan University, Chengdu, 610041, China.; 3 Guangdong Key Laboratory of Liver Disease Research, Key Laboratory of Liver disease biotherapy and Translational Medicine of Guangdong Higher Education Institutes, the Third Affiliated Hospital of Sun Yat-sen University, Guangzhou, Guangdong, 510630, China.

**Keywords:** hepatocellular carcinoma, liver transplantation, albumin-to-alkaline phosphatase ratio, prognosis

## Abstract

**Background**: The albumin-to-alkaline phosphatase ratio (AAPR) is a newly developed index which was used to predict prognosis of HCC patients. However, its prognostic role in HCC patients undergoing liver transplantation (LT) remains unclear. This study aimed to investigate the correlation between AAPR and prognosis of these patients.

**Methods**: A total of 210 patients who underwent LT from January 2003 to January 2014 were retrospectively analyzed (149 for discovery and 61 for validation). Univariate and multivariate analyses were performed to determine the discriminative ability of the AAPR in predicting long-term survival. The area under the receiver operating characteristic (AUC) was calculated to compare the accuracy of different factors.

**Results**: Patients with high AAPR level were associated with less ascites rate (30.6% versus 53.2%, *P*=0.033) as well as more frequencies of Child-Pugh class A (73.6% versus 35.1%, *P*=0.001). Univariate and multivariate analyses suggested the AAPR was independent prognostic factor in predicting overall survival (HR: 0.585, 95% CI: 0.363-0.941, *P*=0.027). Validation cohort confirmed prognostic value of AAPR. Subgroup analysis demonstrated that reduced AAPR level was associated with worse prognosis in HCC patients categorized in Child-Pugh class A (*P*=0.029). The AUCs of the AAPR were 0.710 and 0.744 in predicting 3-year and 5-year survival outcomes, respectively.

**Conclusions**: The study showed in two independent cohorts of HCC patients treated by LT that elevated AAPR was associated with better OS. As a low-cost routine laboratory test, it should be considered as biomarker in the clinical management of HCC.

## Introduction

Hepatocellular carcinoma (HCC) is one of the most common malignancies and the third leading cause of malignancy-related mortality worldwide 1-4. It gives rise to nearly 745500 deaths worldwide in 2012. Multiple treatment strategies are available for HCC, but surgical resection and liver transplantation (LT) are considered to be the most effective curative treatment modalities 5. Hepatectomy removes solitary lesions in patients with preserved liver function, while LT provides both an oncologic resection as well as replacement of a diseased liver 6. Moreover, in patients with underlying cirrhotic liver disease, LT is a more appropriate therapeutic strategy 7. However, its application is restricted by the shortage of available donor livers 8. Therefore, it is critical to select appropriate candidates for LT. The criteria of HCC candidates' selection varied in different transplantation centers all over the world. The Milan criteria, which were proposed by Mazzaferro et al in 1996, are considered as strict standard and restrict LT for HCC patients with a single tumor no more than five cm in diameter, or up to three tumors, none of which exceed three cm 7, 9 . Patients with HCC meeting Milan criteria were associated with favorable outcomes after LT. Recently, multiple expanded criteria have been introduced, such as the Hangzhou criteria and the University of California, San Francisco (UCSF) criteria 10, 11. The Hangzhou criteria were proposed by Zheng et al in 2008 in consideration of the fact that the main etiology of HCC in China to encompass patients with and more numerous larger tumors 12. The Hangzhou criteria restrict LT for HCC patients with a total tumor size no more than eight cm in diameter, or a total tumor diameter more than eight cm with a histopathologic grade of well or moderate differentiation as well as a preoperative alpha-fetoprotein (AFP) level no more than 400 ng/mL. HCC patients within the Hangzhou criteria were supposed to gain satisfactory survival after LT according to several centers reproduced the model 13, 14. However, merely the criteria for HCC candidates' selection is not optimal. The combination selection criteria with staging systems or serum biomarkers will be more beneficial to predict prognosis of HCC patients undergoing LT.

Several scoring systems have been investigated to predict the clinical prognosis of HCC patients underwent LT and to guide therapeutic regimen. The American Joint Committee on Cancer (AJCC) TNM staging system, the Barcelona Clinic Liver Cancer (BCLC) system and the Japan Integrated Staging (JIS) are the most widely used ones in predicting survival outcomes of patients with HCC^15^-^17^. Moreover, serum parameters, such as circulating immune-inflammatory cells like neutrophils and lymphocytes, alpha-fetoprotein (AFP), albumin (ALB), bilirubin, and alkaline phosphatase (ALP), have also been investigated for their potential prognostic predicting value 18-21. Increasing attention has been paid to the inflammation-based prognostic models in recent years, such as neutrophil-to-lymphocyte ratio (NLR), platelet-to-lymphocyte ratio (PLR) and the systemic immune-inflammation index (SII) 9, 22, 23. The prognosis of patients with HCC depends not only on tumor burden (tumor size, number, portal vein thrombosis and extrahepatic spread) but also underlying liver function 24. The Child-Pugh (CP) classification and the Albumin-Bilirubin (ALBI) grade, introduced to assess liver function initially, have been verified to be prognostic predictors for HCC patients. Recently, a newly-presented biomarker named albumin-to-alkaline phosphatase ratio (AAPR) was used to predict survival of HCC patients undergoing curative resection and palliative therapy 25. In advanced HCC patients without receiving any standard anti-cancer therapies, AAPR could also serve as a potentially valuable prognostic index 26. Nevertheless, its potential prognostic value in patients with HCC undergoing liver transplantation is still unclear.

In the present study, we analyzed a cohort of patients with HCC meeting Hangzhou criteria to explore the correlation between AAPR and clinicopathological characteristics, and its value in predicting survival outcomes.

## Material and methods

### Study population

We retrospectively enrolled patients who underwent LT from January 2003 to May 2013 as the discovery cohort. The validation cohort included patients treated by LT from June 2013 to January 2014. All the included patients were newly diagnosed as HCC meeting Hangzhou criteria and admitted in the Third Affiliated Hospital of Sun Yat-sen University. Patients were excluded if they received liver resection, radiofrequency ablation, transarterial chemoembolization, chemotherapy or other anti-cancer therapies before LT 27. Patients were also excluded if gastrointestinal hemorrhage happened prior to LT. All eligible patients or their relative freely gave written informed consent. This study was approved by the ethics committee of the Third Affiliated Hospital of Sun Yat-sen University, in accordance with the guidelines of the 1975 Declaration of Helsinki 28.

### Data collection and follow-up

The following data of pretreatment clinical information were reviewed and collected from the hospital handwritten or electronic medical records: patients' basic information, medical history and laboratory examination including serum alanine transaminase (ALT), aspartate transaminase (AST), ALB, total bilirubin (TBIL) and ALP were collected at the time of preoperative, then were analyzed for baseline evaluation. Tumor-related clinicopathological characteristics including preoperative AFP level, differentiation, the number of tumor nodules, maximum tumor diameter and metastasis were also acquired. The definition of microvascular invasion (MVI) is based on the guideline of the HCC standardized pathological diagnosis 29. The AAPR was calculated from dividing the ALB level by serum ALP level, where ALB was in g/L and ALP in U/L 25. Patients were followed up according to National Comprehensive Cancer Network (NCCN), regularly contrast-enhanced ultrasonography per month at first year, then every 3 months for 2 years, and then every 6 months thereafter. Besides, we contact those who determined not to go back to the hospital to reexamination through telephone follow-up survey. All the patients were followed up for 5 years at the endpoint.

### Statistical analysis

The software of SPSS (version 22.0), MedCalc (version 15.2.2.0) and Graphpad Prism (version 5.0) were used to perform statistical analyses. We used Pearson's chi square test and student's *t*-test to investigate the correlation of categorical and continuous variables to AAPR level respectively. The primary endpoint was overall survival (OS), defined as the duration from surgery to HCC-associated mortality. The secondary endpoint was recurrence-free survival (RFS), defined as the duration from surgery to recurrence. Receiver-operating characteristic (ROC) curves were applied to determine the optimal cut-offs as Youden index attained maximum value at 5 years posttransplant. Patients were categorized by AAPR level. Kaplan-Meier method was used to perform survival analysis for the groups with different cut-off values, and their differences were tested with log-rank test. Those clinicopathological parameters with *P*<0.05 in the univariable Cox proportional hazards regression were considered for generating multivariable Cox regression (enter method) to identify potential independent prognostic factors for OS of HCC patients underwent LT. A two-tailed *P* value less than 0.05 was considered statistically significant. Subgroup analysis was conducted in patients with liver function of CP class A. Kaplan-Meier curves were introduced to analyze the correlation between AAPR level and OS as well as RFS in HCC patients with proper liver function.

## Results

### Patient characteristics

The baseline demographic and clinical characteristics of discovery and validation cohort were showed in Table 1. The entire cohort contained a total of 210 HCC patients treated by LT with mean age of 51.47 (198 male). There was no statistical difference between discovery and validation cohort in baseline characteristics.

In the discovery cohort, a total of 149 patients with newly diagnosed HCC were included with a mean age of 51.26. Most of the included patients were male (141, 94.6%). Additionally, 138 patients were associated with HBV infection. Among the total sample set, 80 patients with liver function of Child A grade, 63 patients were associated with pretreatment ascites. As for laboratory test, ALT, AST, TBIL, ALB and ALP were 52.42±39.02, 65.79±43.74, 65.73±137.42, 37.54±5.46, and 102.87±25.25, respectively. 56 patients were associated with tumor size over 5 cm, 27 patients with tumors of well-differentiation, 24 with MVI and 56 patients with tumor-node-metastasis (TNM) stage III. 3-year survival rate and 5-year survival rate for total sample size were 61.7% and 45.6%.

In the validation cohort, 61 patients with a mean age of 52.15 (57 male). 55 patients were associated with HBV infection. The CP classification were: A (n=30, 49.5%), B&C (n=31, 50.5%). 26 patients were associated with pretreatment ascites. As for laboratory test, ALT, AST, TBIL, ALB and ALP were 51.49±33.24, 69.84±51.31, 83.48±161.17, 37.59±5.19, and 114.97±34.92, respectively. 21 patients were associated with tumor size over 5 cm, 17 patients with tumors of well-differentiation, 14 with MVI and 30 patients with tumor-node-metastasis (TNM) stage III. 3-year survival rate and 5-year survival rate for total sample size were 61.7% and 45.6%.

### Correlation between patient clinicopathological characteristics with AAPR level

The optimal cut-off values with the maximum Youden index value were 0.38 and 112 for AAPR and ALP, respectively. Consequently, in the discovery cohort, 72 patients with AAPR value more than 0.38 were categorized into high AAPR group and 77 patients in low group. In validation cohort, 26 patients were classified into AAPR high group while 35 patients in low group. The correlation between AAPR level with clinicopathological characteristics were shown in Table 2. Higher 3-year survival rate (72.2% vs 51.9% in discovery cohort and 80.8% vs 51.4% in validation cohort, respectively) and 5-year survival rate (61.1% vs 31.2% in discovery cohort and 73% vs 42.9% in validation cohort, respectively) were observed in high AAPR group.

**Figure 1:**
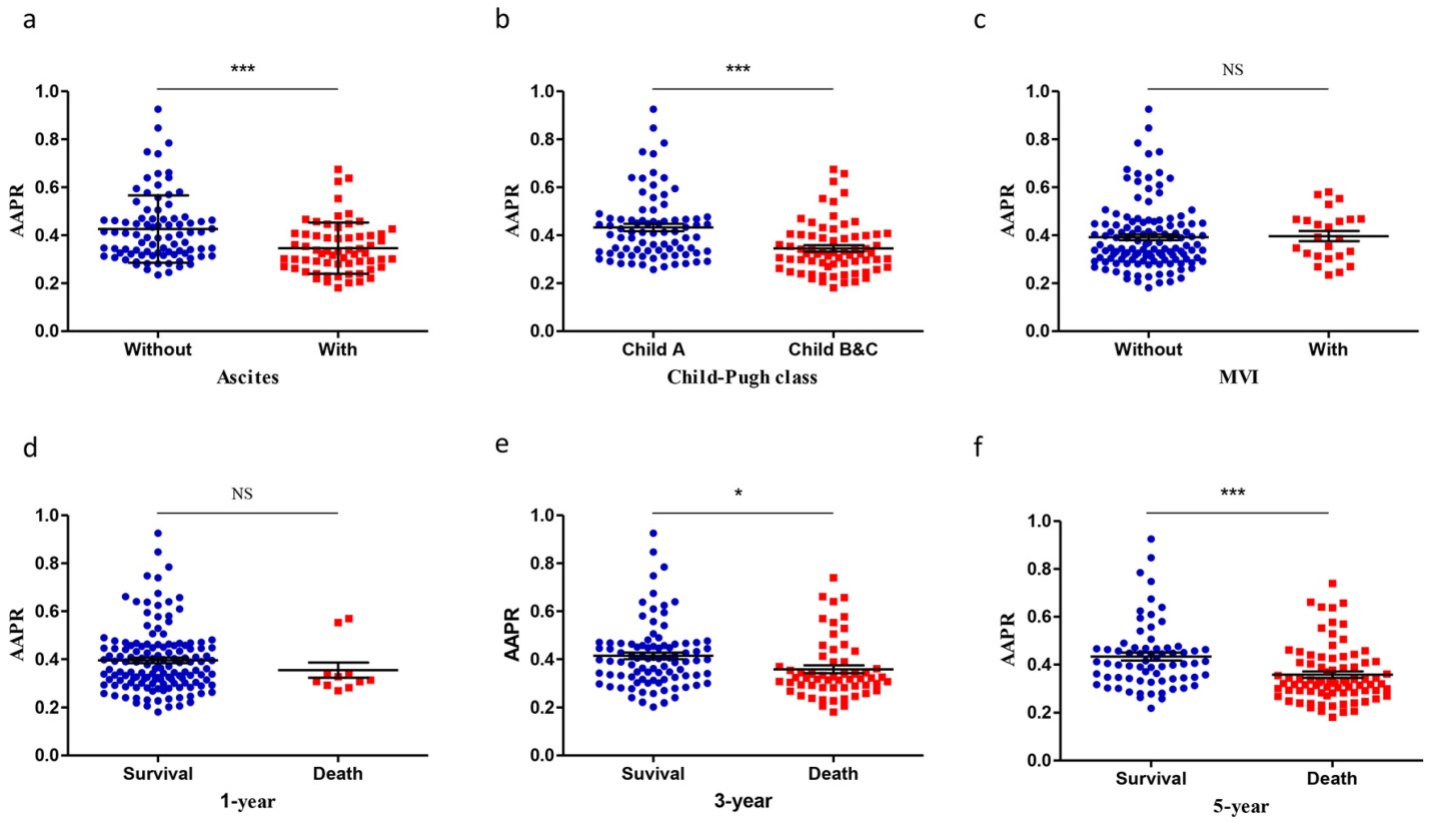
Scatter plots showing the AAPR values in different subgroups categorized by: (a) pretreatment ascites; (b) Child-Pugh class; (c) pretreatment MVI; (d) 1-year survival state; (e) 3-year state and (f) 5-year state. The means and standard deviations for each group were signified by the black lines within the scatter plots. AAPR: albumin-to-alkaline phosphatase ratio; MVI: microvascular invasion. *: *P*<0.05; **: *P*<0.01; ***: *P*<0.001.

In the discovery cohort, a higher AAPR level was observed in patients without ascites (0.426±0.015 vs 0.347±0.013, *P*<0.001, Figure 1a). Patients with Child A grade liver function were associated with higher AAPR value (0.433±0.015 vs 0.346±0.013, P<0.001, Figure 1b). No significant difference was found between patients with MVI and those without (Figure 1c). Patients stratified in 3-year survival group showed higher AAPR value (0.414±0.014) than death group (0.358±0.016) (*P*=0.011, Figure 1e). Patients were associated with lower AAPR level in 5-year death group (0.358±0.013) than survival group (0.433±0.017) (P<0.001, Figure 1f).

### Survival analysis

**Figure 2:**
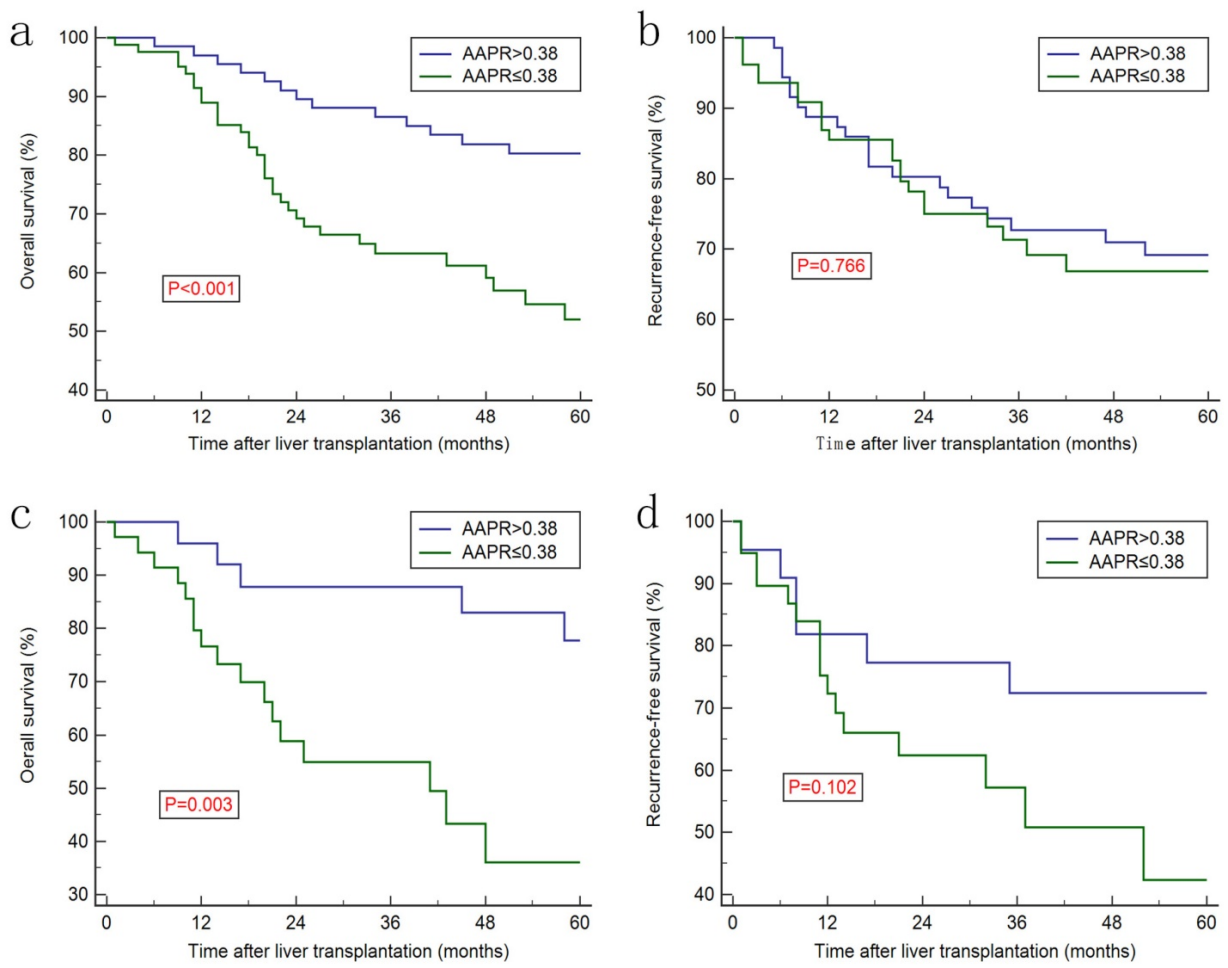
Kaplan-Meier curves for overall survival and recurrence-free survival stratified by AAPR level in discovery cohort (a & b) and validation cohort (c &d).

**Figure 3:**
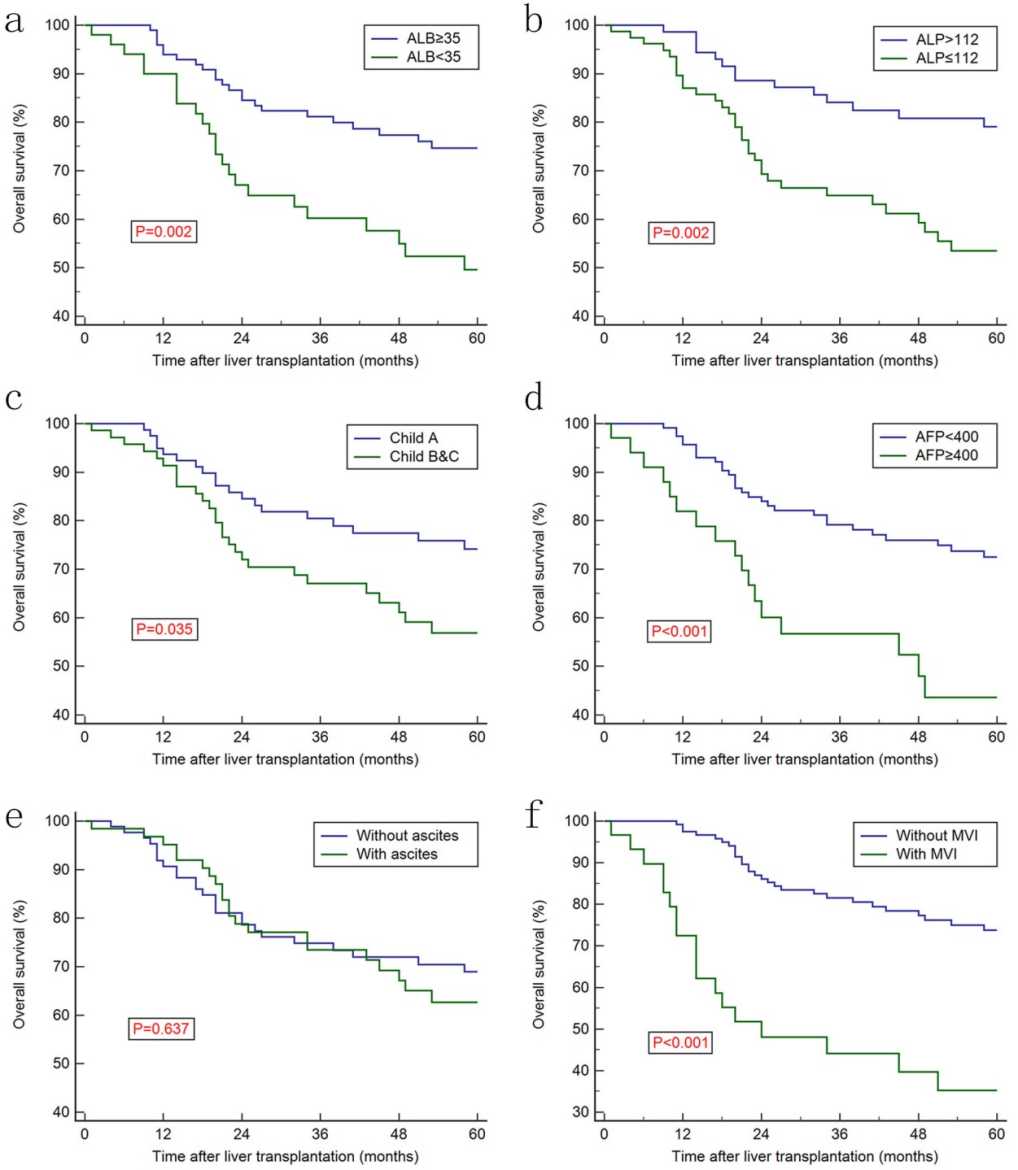
Kaplan-Meier survival curves for overall survival stratified by: ALB of 35 g/dl (a), (d) the optimal cut-off value of ALP at 112 (b), Child-Pugh class(c), AFP (d), pretreatment ascites status (e) and MVI status (f). AAPR: albumin-to-alkaline phosphatase ratio; ALB: albumin; ALP: alkaline phosphatase; MVI: microvascular invasion.

**Figure 4:**
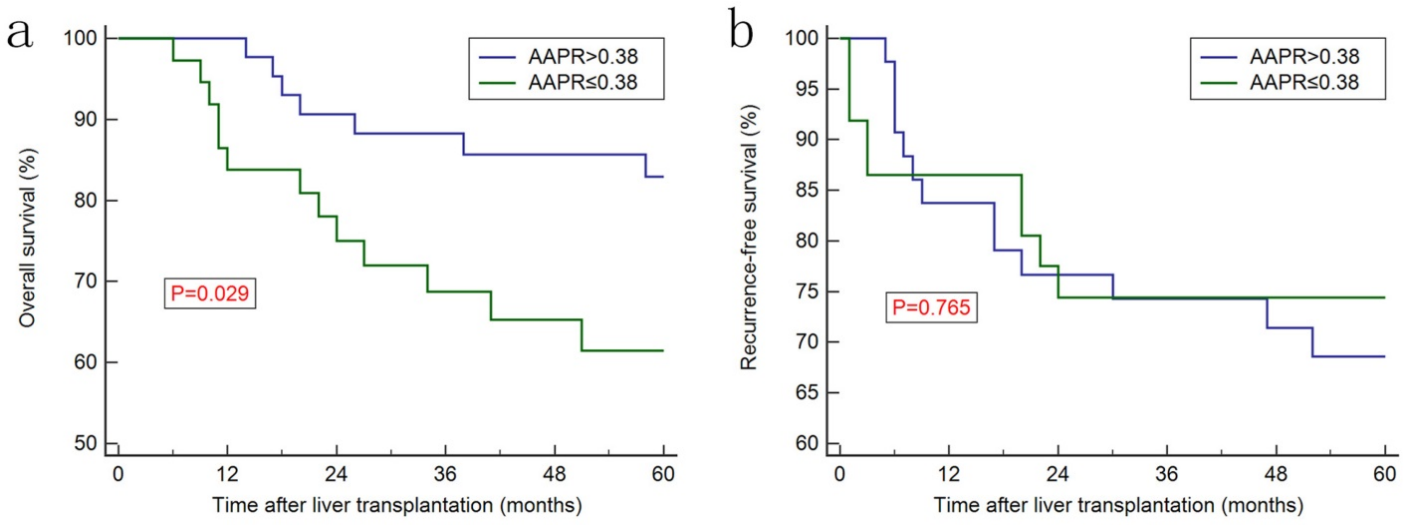
Kaplan-Meier curves for overall survival (a) and recurrence-free survival (b) in subgroup of patients with liver function of Child-Pugh class A.

Increased AAPR level was associated with better prognosis in discovery cohort (Figure 2a) and in the validation cohort (Figure 2c), whereas no significant difference was observed regarding recurrence-free survival (RFS) between two groups (Figure 2b and 2d). Furthermore, Kaplan-Meier curves showed that the ALB, ALP, AFP, CP classification and MVI were prognostic factors for OS (Figure 3). Univariate analysis revealed that CP class [hazard ration (HR): 1.858, 95% confidence interval (CI): 1.037-3.329, *P*=0.035], ALB (HR: 0.419, 95% CI: 0.223-0.790, *P*=0.002), ALP (HR: 2.625, 95% CI: 1.471-4.686, P<0.001), AFP (HR: 2.626, 95% CI: 1.243-5.551, *P*<0.001), TNM stage (HR: 2.370, 95% CI: 1.496-3.754, *P*<0.001) and AAPR (HR: 0.505, 95% CI: 0.325-0.784, *P*<0.001) were associated with significant difference. However, after conducting multivariate analysis, only AAPR was identified to be independent prognostic factor for HCC patients underwent LT (HR: 0.585, 95% CI: 0.363-0.941, *P*=0.027) (Table 3). In subgroup analyses, Kaplan-Meier confirmed the overall outcomes that patients with high AAPR level were associated longer OS compared to those with low level (*P*=0.029, Figure 4a) in patients with CP class A. The RFS remained comparable between two groups (*P*=0.765, Figure 4b).

### Accuracy for AAPR in predicting OS

**Figure 5:**
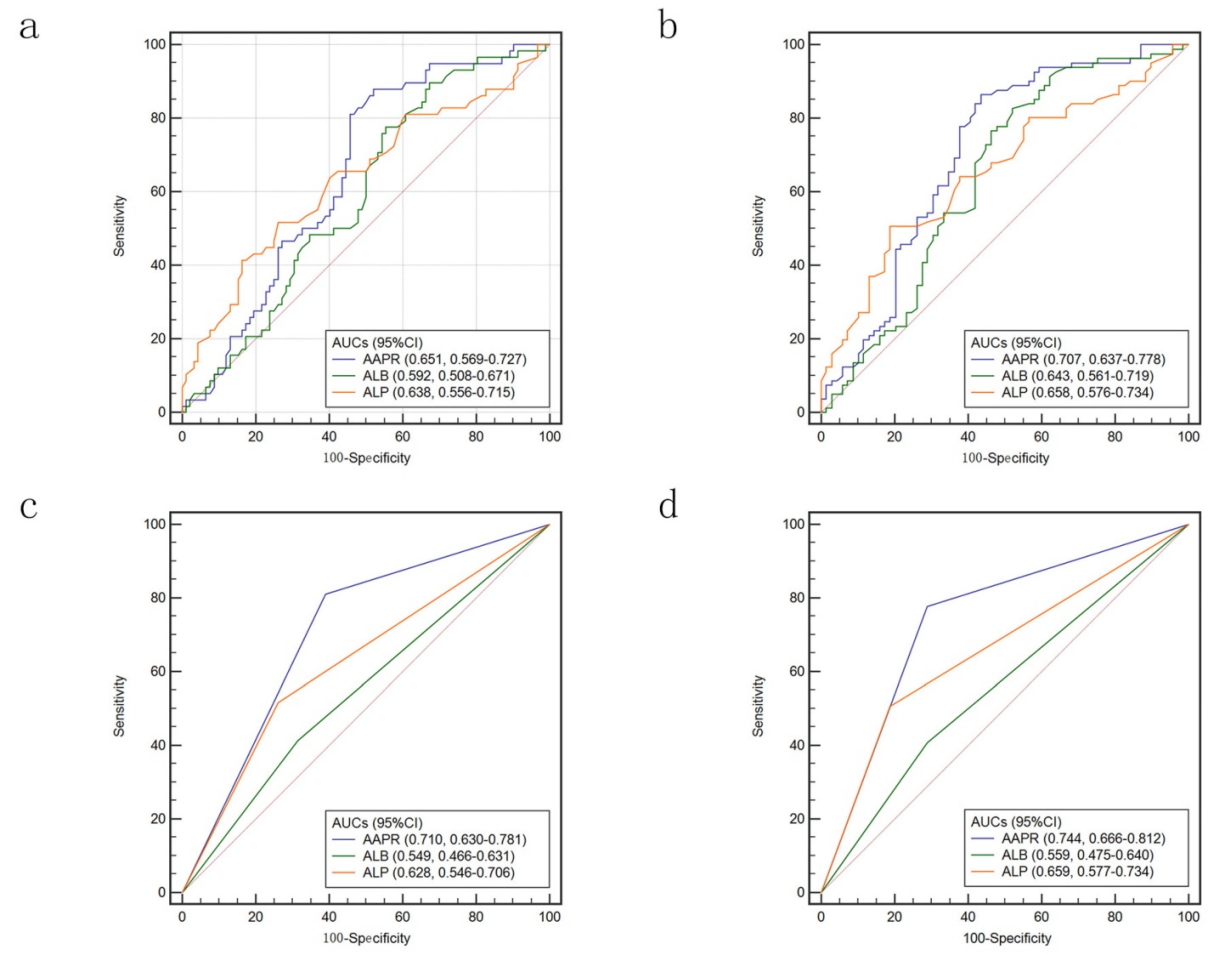
Comparison of AUCs for AAPR, ALB and ALP: calculated as continuous variables in predicting 3-year survival (a) and 5-year-survival (b); calculated as categorized variables in predicting 3-year survival (c) and 5-year-survival (d). AUC: area under the receiver operating characteristic; AAPR: albumin-to-alkaline phosphatase ratio; ALB: albumin; ALP: alkaline phosphatase.

ROC curves were used to compare the accuracy of AAPR, ALB and ALP in predicting prognosis of HCC patients underwent LT. When we used continuous variable to conduct ROC curves, the area under the receiver operating characteristic (AUC) of AAPR was 0.651 for 3-year survival and 0.707 for 5-year survival (Figure 5a & 5b). The AUCs were higher than that of ALB and ALP. While the variables were treated as categorical variables, both AUCs were larger. For predicting 3-year survival, the AUCs were 0.710, 0.549 and 0.628 for AAPR, ALB and ALP, respectively (Figure 5c). When 5-year survival was set as endpoint, the AUCs were 0.744, 0.559 and 0.659 for AAPR, ALB and ALP, respectively (Figure 5d).

## Discussion

HCC remains one of the most complicated abdominal malignancies, and its therapeutic strategy remains a challenge 30. Various scoring systems were developed to evaluate the prognosis and guide clinical management of HCC patients. However, there is still lack of a universally accept scoring system for classification HCC 31, 32. Liver function is an easily accessible laboratory parameter, which is a necessarily detected index for patients with HCC. Child-Pugh classification and the ALBI score, concerning liver function, were suggested to be prognostic systems for HCC patients underwent liver resection and other standard anti-cancer therapies 33-35. Serum ALB, a kind of protein synthesized in liver, is an indictor reflects the protein synthetic capability. Moreover, it remains a modulator for inflammatory response, which is essential in retardation of HCC 36. Recent studies have demonstrated the ability of ALB in stabilizing cell proliferation and exerting antioxidant reaction for anti-carcinogenesis 37, 38. Therefore, it was considered to be an independent prognostic factor for HCC patients and a variable integrated into several scoring systems like the ALBI score and the Chinese University Prognostic Index (CUPI) system 39. The serum ALP, another one of the most routinely detected parameters in laboratory test, is a hydrolase enzyme and exists throughout the body, especially concentrates in the liver, bones, kidney and placenta with multiple isoforms. Several studies have reported that the ALP level increases during childhood and other diseases such as hepatic diseases, osteomalacia, and bone tumor 40. Several evidences indicated that ALP played important roles in strengthening cancer cell proliferation, vascular invasion and distant metastasis 41. In addition, ALP has also been reported as an independent factor for HCC patients which was correlated with cirrhosis and prognosis 42. The AAPR was introduced by Chan et al initially to predict prognosis of HCC patients who underwent surgical resection and palliative therapy 25. Cai et al investigated its prognostic value in advanced HCC patients who did not receive any standard anti-cancer treatments 26. Nonetheless, it remained unclear whether AAPR could accurate predict the prognosis of HCC patients underwent LT.

The present study showed that pre-transplantation AAPR was an independent prognostic index for HCC patients within Hangzhou criteria. However, there was no significant correlation between the AAPR and RFS of HCC patients underwent LT. The estimated AAPR level was higher in patients with liver function of CP class A and patients without pretreatment ascites to those with CP class B & C and those with ascites. It confirmed the results that patients with high AAPR value were associated with higher frequencies of CP class A as well as ascites compared to those with lower AAPR level. After calculating Youden index, a 0.38 was supposed to be optimal cut-off value. Kaplan-Meier curves indicated that patients with AAPR value of more than 0.38 were associated with longer OS. Univariate and multivariate analyses confirmed that the AAPR was independent prognostic index for HCC patients underwent LT. In subgroup of patients with liver function of CP class A, the AAPR remained its prognostic role in predicting OS.

This was the first study investigated the potential prognostic value of AAPR in HCC patients underwent LT. ALB and ALP are both simple but different and objective variables, which are more easily applied in clinical practice. However, ALB and ALP have never been put together to evaluate their combined prognostic significance. Hence, we introduced a novel and simple index, AAPR. The AAPR is a powerful prognostic indicator with the highest c-index and χ^2^ (by LR test) among other liver biochemical parameters 25. In our study, comparison of ROC curves implied that AAPR preceded ALB or ALP alone as a more accurate prognostic index for OS in HCC patients underwent LT. Unlike other serodiagnosis or iconographical detections, it was a novel index readily derived from a simple low-cost routine blood test which would not increase the total medical costs. Furthermore, our study analyzed the correlation between AAPR level with clinicopathological characteristics of patients. Further researches were in need to confirm our primary outcomes and elucidate the potential molecular mechanisms.

However, there were several limitations that warrant consideration when interpreting our findings. Firstly, the present study was retrospectively designed, which would be associated with potential selection bias though strict criteria were developed in population enrollment. Secondly, all the patients were Chinese from single center and most of them were associated with chronic HBV infection. Furthermore, the sample size was not large enough to perform subgroup analysis. Additionally, a validation cohort was in need to confirm our main results. Finally, the underlying molecular mechanism of the correlation between the AAPR with prognosis of HCC should be further investigated.

## Conclusions

In summary, the present study showed in two independent cohorts of HCC patients treated with LT suggested that increased AAPR was associated with better prognosis. Multivariable analysis identified AAPR as a potential prognostic factor for OS. Subgroup analysis of patients categorized in CP class A revealed the AAPR played a prognostic role in predicting OS. As a low-cost routine laboratory test, it provided additional prognostic information for tumor scoring systems and could be viewed as biomarker in the clinical management of HCC.

## Figures and Tables

**Table 1:** Characteristics of entire cohort of HCC patients underwent liver transplantation.

Variables	All patients (*n*=210)	Discovery (*n*=149)	Validation (*n*=61)	*P* Value
Age (year)	51.47±9.76	51.26 ±9.96	52.15±9.28	0.524
Gender (M/F)	198/12	141/8	57/4	0.729
HBsAg (+/-)	193/17	138/11	55/6	0.547
Child-Pugh class				
A	110	80	30	0.524
B&C	100	69	31	
Ascites (+/-)	89/121	63/86	26/35	0.513
ALT (IU/L)	52.21±37.28	52.42±39.02	51.49±33.24	0.859
AST (IU/L)	67.53±46.59	65.79±43.74	69.84±51.31	0.648
TBIL (μmol/L)	70.75±144.22	65.73±137.42	83.48±161.17	0.415
ALB (g/L)	37.55±5.37	37.54±5.46	37.59±5.19	0.981
ALP (U/L)	106.38±28.84	102.87±25.25	114.97±34.92	0.783
AFP (≥400/<400)(ng/dL)	45/165	29/120	16/45	0.583
Tumor size (>5cm/≤5cm)	77/133	56/93	21/40	0.628
Differentiation (Well/Moderate)	44/166	27/122	17/44	0.135
MVI (+/-)	38/172	24/125	14/47	0.070
TNM stages (Ⅰ-Ⅱ/Ⅲ)	134/76	93/56	41/20	0.503
MELD score	9.29±7.05	8.94±7.16	10.14±6.79	0.469
1-year survival	189/21	138/11	51/10	0.059
3-year survival	131/79	92/57	39/22	0.726
5-year survival	102/108	68/81	34/27	0.060

AAPR, albumin-to-alkaline phosphatase ratio; M, male; F, female; ALT, alanine aminotransferase; AST, aspartate aminotransferase; TBIL, total bilirubin; ALB, albumin; ALP, alkaline phosphatase; AFP, alpha-fetoprotein; MVI, microvascular invasion; TNM, tumor-node-metastasis; MELD score, model for end-stage liver diseases score. Data were expressed as numbers of patients or mean ± SD.

**Table 2:** Correlation between AAPR and clinicopathological characteristics in discovery and validation cohorts.

Variables	Discovery			Validation		
	AAPR>0.38 (*n*=72)	AAPR≤0.38 (*n*=77)	*P* Value	AAPR>0.38 (n=26)	AAPR≤0.38 (n=35)	*P* Value
Age (year)	49.89±9.90	52.55±9.92	0.104	50.35±8.46	53.49±9.75	0.194
Gender (M/F)	70/2	71/6	0.278	24/2	33/2	0.762
HBsAg (+/-)	69/3	69/8	0.212	26/0	29/6	0.026
Child-Pugh class						
A	53	27	0.001	15	16	0.363
B&C	19	50		11	19	
Ascites (+/-)	22/50	41/36	0.033	10/16	16/19	0.579
ALT (IU/L)	51.64±36.41	53.14±41.55	0.815	46.88±24.74	54.91±38.36	0.355
AST (IU/L)	63.08±35.15	68.32±50.59	0.467	61.04±30.35	76.37±62.17	0.252
TBIL (μmol/L)	53.76±109.58	75.61±155.96	0.337	73.23±151.79	92.19±170.56	0.113
ALB (g/L)	41.60±3.80	34.23±4.23	<0.001	39.61±4.98	36.09±4.88	0.007
ALP (U/L)	83.85±17.68	120.65±16.92	<0.001	79.24±10.92	141.52±19.04	<0.001
AFP (≥400/<400)(ng/dL)	12/60	17/60	0.418	6/20	10/25	0.636
Tumor size (>5cm/≤5cm)	27/45	29/48	1.000	7/19	14/21	0.296
Differentiation (Well/Moderate)	17/55	10/67	0.135	11/15	6/29	0.03
MVI (+/-)	13/59	11/66	0.657	8/18	6/29	0.217
TNM stages (Ⅰ-Ⅱ/Ⅲ)	47/25	46/31	0.503	18/8	23/12	0.777
MELD score	8.50±5.39	9.35±8.51	0.469	9.50±6.20	10.63±7.26	0.526
1-year survival	70/2	68/9	0.057	25/1	26/9	0.022
3-year survival	52/20	40/37	0.012	21/5	18/17	0.018
5-year survival	44/28	24/53	<0.001	19/7	15/20	0.018

AAPR, albumin-to-alkaline phosphatase ratio; M, male; F, female; ALT, alanine aminotransferase; AST, aspartate aminotransferase; TBIL, total bilirubin; ALB, albumin; ALP, alkaline phosphatase; AFP, alpha-fetoprotein; MVI, microvascular invasion; TNM, tumor-node-metastasis; MELD score, model for end-stage liver diseases score. Data were expressed as numbers of patients or mean ± SD.

**Table 3:** Independent prognostic factors for overall survival by the univariate and multivariate Cox proportional hazards regression model for discovery cohort.

Variables	Univariate			Multivariate		
	HR	95% CI	*P*	HR	95% CI	*P*
Age	1.007	0.978-1.037	0.652			
Gender (F/M)	0.731	0.177-3.018	0.665			
HBsAg (+/-)	0.557	0.268-1.156	0.116			
Child-Pugh class	1.858	1.037-3.329	0.035	1.418	0.884-2.277	0.148
Ascites (+/-)	1.149	0.640-2.063	0.637			
ALT	0.999	0.993-1.005	0.651			
AST	1.001	0.995-1.006	0.842			
TBIL	1.001	0.999-1.002	0.250			
ALB	0.419	0.223-0.790	0.002			
ALP	2.625	1.471-4.686	<0.001			
AFP (≥400/<400)	2.626	1.243-5.551	<0.001	1.574	0.949-2.613	0.079
Differentiation (Moderate/Well)	1.263	0.698-2.287	0.440			
Tumor size (>5/≤5)	1.355	0.872-2.103	0.176			
MVI (+/-)	4.029	1.742-9.325	<0.001	1.934	0.668-5.594	0.224
TNM stages (Ⅲ/Ⅰ-Ⅱ)	2.370	1.496-3.754	<0.001	1.387	0.762-2.524	0.285
MELD score	1.023	0.994-1.052	0.118			
AAPR	0.505	0.325-0.784	<0.001	0.585	0.363-0.941	0.027

AAPR, albumin-to-alkaline phosphatase ratio; M, male; F, female; ALT, alanine aminotransferase; AST, aspartate aminotransferase; TBIL, total bilirubin; ALB, albumin; ALP, alkaline phosphatase; AFP, alpha-fetoprotein; MVI, microvascular invasion; TNM, tumor-node-metastasis; MELD score, model for end-stage liver diseases score. HR, hazard ratio; CI, confidence interval.

## Abbreviations

AAPR: albumin-to-alkaline phosphatase ratio
LT: liver transplantation
AAPR: albumin-to-alkaline phosphatase ratio
AUC: receiver operating characteristic
UCSF: University of California, San Francisco
AFP: alpha-fetoprotein
AJCC: American Joint Committee on Cancer
BCLC: Barcelona Clinic Liver Cancer
JIS: Japan Integrated Staging
ALB: albumin
ALP: alkaline phosphatase
NLR: neutrophil-to-lymphocyte ratio
SII: systemic immune-inflammation index
CP: Child-Pugh
ALBI: Albumin-Bilirubin
MVI: microvascular invasion
NCCN: National Comprehensive Cancer Network
OS: overall survival
RFS: recurrence-free survival
CUPI: Chinese University Prognostic Index
